# Creutzfeldt-Jakob disease after COVID-19: infection-induced prion protein misfolding? A case report

**DOI:** 10.1080/19336896.2022.2095185

**Published:** 2022-07-03

**Authors:** Andrea Bernardini, Gian Luigi Gigli, Francesco Janes, Gaia Pellitteri, Chiara Ciardi, Martina Fabris, Mariarosaria Valente

**Affiliations:** a Clinical Neurology Unit, Santa Maria della Misericordia University Hospital, Udine, Italy; b Department of Medicine, University of Udine, Udine, Italy; c Neuroradiology Unit, Santa Maria della Misericordia University Hospital, Udine, Italy; d Institute of Clinical Pathology, Santa Maria della Misericordia University Hospital, Udine, Italy

**Keywords:** Creutzfeldt-Jakob disease, COVID-19, prion, neuroinflammation, neurodegeneration, protein misfolding

## Abstract

Creutzfeldt-Jakob disease (CJD) is a rare, fatal disease presenting with rapidly progressive neurological deficits caused by the accumulation of a misfolded form (PrPSc) of prion protein (PrPc). Coronavirus disease 2019 (COVID-19) is a primarily respiratory syndrome caused by the severe acute respiratory syndrome coronavirus 2 (SARS-CoV-2); many diverse neurological complications have been observed after COVID-19. We describe a young patient developing CJD two months after mild COVID-19. Presenting symptoms were visuospatial deficits and ataxia, evolving into a bedridden state with preserved consciousness and diffuse myoclonus. Diagnostic work-up was suggestive of CJD. The early age of onset and the short interval between respiratory and neurological symptoms might suggest a causal relationship: a COVID-19-related neuroinflammatory state may have induced the misfolding and subsequent aggregation of PrPSc. The present case emphasizes the link between neuroinflammation and protein misfolding. Further studies are needed to establish the role of SARS-CoV-2 as an initiator of neurodegeneration.

## Introduction

Creutzfeldt-Jakob disease (CJD) is a rare, fatal disease presenting with rapidly progressive neurological deficits; it is caused by the accumulation of neurotoxic aggregates of misfolded prion protein (PrPSc) [1]. Sporadic CJD (sCJD) represents 85% of all CJD cases; the mean age at onset of sCJD is 67 years [1]. Coronavirus disease 2019 (COVID-19) is a primarily respiratory syndrome caused by the SARS-CoV-2 coronavirus [2], whose high infectivity led to a still ongoing worldwide pandemic. Many diverse neurological complications have been observed in COVID-19 cases, with hyposmia, cerebrovascular diseases, headache, cognitive deficits and Guillain-Barré syndrome being the most common [2]. Here we describe a case of sCJD following COVID-19.

## Results

### Clinical summary

**Figure 1.**
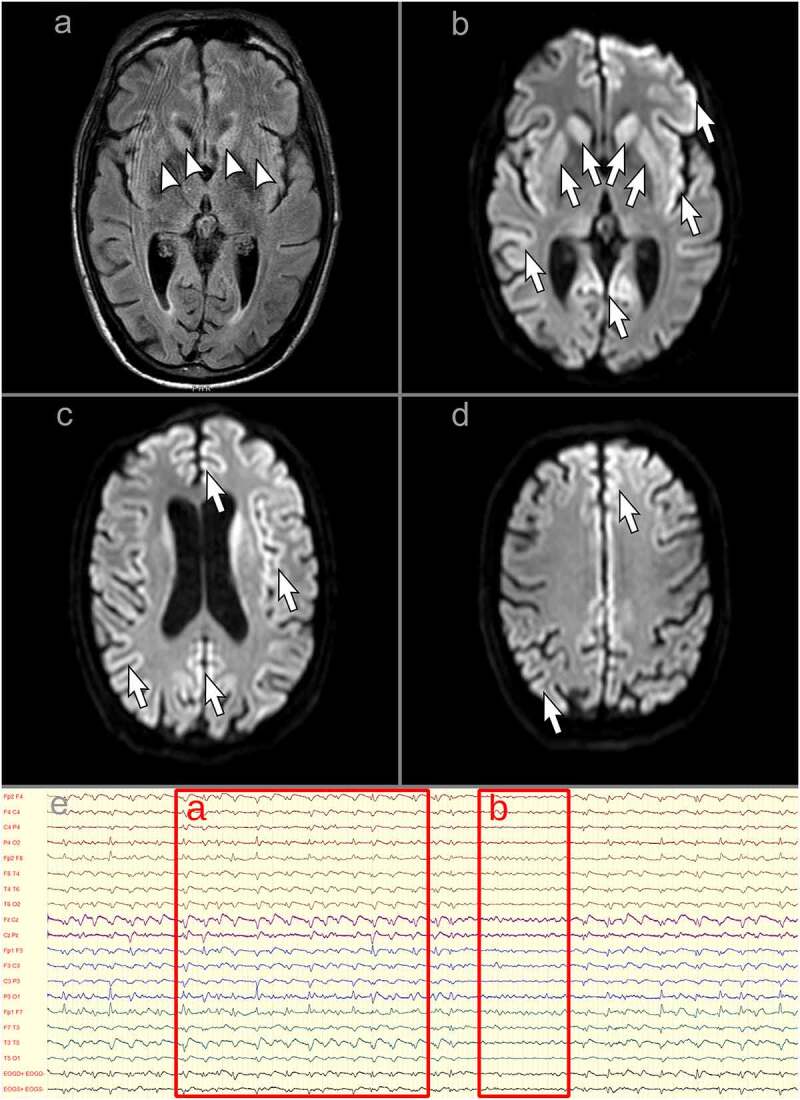
A-D: magnetic resonance imaging of the brain showing typical features of Creutzfeldt-Jakob disease: hyperintensity of the caudate nuclei and putamina on fluid-attenuated inversion recovery imaging (panel a, arrowheads) and diffusion restriction within the bilateral striatum, right fronto-temporo-insular cortex, left fronto-parietal cortex and bilateral occipital cortex on diffusion-weighted imaging (panels b-d, arrows). e: electroencephalographic recording showing typical features of Creutzfeldt-Jakob disease. a longitudinal montage is depicted. Subcontinuous generalized triphasic periodic sharp-wave complexes can be seen on this segment, with a 1–2 Hz discharge rate (a); during the short interruptions in periodic sharp-wave complex firing, a diffusely slowed background activity in the theta range can be observed (b).

A previously healthy man in his early forties developed mild COVID-19 in March 2021, with persistent fatigue and transient hyposmia and hypogeusia being the only reported symptoms. His previous history was significant for atopic dermatitis and saphenous vein stripping due to chronic venous insufficiency. His family history was reported to be negative for neurological diseases. However, his father died at 33 years of age, apparently due to myocardial infarction, while his mother had been rescued from Shoah and adopted in her infancy (she was reported to be of Spanish origin, possibly Sephardim). In the second half of May 2021 he started seeing black shadows when closing his eyes, followed by dizziness, difficulty reading and worsening of balance. In June 2021 he noticed loss of coordination of the left arm. Due to gradual progression, he was admitted to a local neurological ward in late June: the neurological exam showed ataxia of the left limbs, right-beating nystagmus and absent lower-limb reflexes. Brain Magnetic Resonance Imaging (MRI) was reportedly normal; lumbar puncture showed normal cerebrospinal fluid (CSF) protein and cells and absence of oligoclonal bands. Serological testing was negative for onconeural antibodies, neuronal surface antibodies, anti-ganglioside antibodies, Treponema pallidum, Borrelia, Human Immunodeficiency Virus and Hepatitis B and C Viruses. He was discharged, to be readmitted shortly thereafter due to the appearance of clonic movements of the left hand. A second brain MRI was again reportedly normal, including diffusion-weighted sequences. Electroencephalographic (EEG) recordings showed frequent diffuse theta-delta slowing with epileptiform discharges on the right hemisphere. Carbamazepine proved ineffective and was therefore replaced with valproic acid, which was in turn substituted with levetiracetam due to excessive drowsiness. Nevertheless, symptoms progressed and repeat EEGs showed an increase in slow activity and generalized discharges described as spike-and-wave. A second onconeural and neuronal surface antibody panel was negative, as well as antinuclear and anti-neutrophil cytoplasmic antibodies. A total-body Positron Emission Tomography-Computed Tomography was normal. A trial of intravenous immunoglobulin was associated with a transient arrest of progression; in the following days, a 5-day high-dose intravenous methylprednisolone was accompanied with an abrupt worsening of incoordination of the left arm and the appearance of dystonia of both upper limbs. At the end of July 2021, the patient was transferred to our ward. On admission, he was drowsy but responsive and partially oriented, his speech was effortful and telegraphic in English (native language untestable). Eye movements were preserved, although saccadic movements were slow in all directions. He could not sit, stand or walk due to diffuse dystonic posturing, more evident on the left side, and diffuse myoclonus. His left upper limb showed non-stereotyped and irregular involuntary movements. A complete strength testing could not be performed due to the inability to adequately perform voluntary movements; however, no major deficits were noted. Similarly, finger-to-nose and heel-to-shin tests could not be performed due to the dystonic and myoclonic components. A repeat brain MRI showed mild T2 hyperintensity of the caudate nuclei and putamina and diffusion restriction within the right fronto-temporo-insular cortex, left fronto-parietal cortex and bilateral occipital cortex (Figure 1, panels a-d). Prolonged EEG recordings showed diffuse theta slowing with generalized triphasic 1–2 Hz periodic sharp-wave complexes (Figure 1, panel e). As shown in Table 1, an increase in serum interleukin (IL)-1β, IL-6 and IL-8 and neurofilament light chain (NfL) was observed, with normal C-reactive protein and mid-regional proadrenomedullin (MR-ProADM) levels; extended autoimmune and infectious panels were negative. SARS-CoV-2 serology showed positive anti-Receptor Binding Domain (RBD) Spike 1 total antibodies at high titre, with negative IgM antibodies; multiple nasal swabs were negative for SARS-CoV-2. CSF analysis showed normal protein, cells and MR-ProADM, with moderately elevated IL-8 and C-X-C motif chemokine Ligand 10 (CXCL10) and very high NfL levels. A neurodegenerative biomarker profile showed extremely high total Tau levels, slightly reduced beta-amyloid_1-42_ and normal phospho-Tau-181 and beta-amyloid 42/40 ratio. Real-Time Quaking-Induced Conversion (RT-QuIC) CSF testing for PrP was positive.

**Table 1.** Relevant laboratory analyses on blood and cerebrospinal fluid samples.

Feature				Value	Reference range
Inflammation	Pro-adrenomedullin	Serum CSF CSF/blood ratio	0.46 nMol/l 0.55 nMol/l 1.2		<0.56
	Interleukin 1β	Serum CSF	**0.2** pg/ml <0.1 pg/ml		<0.16 <0.1
	Interleukin 6	Serum CSF	**42.5** pg/ml 4.4 pg/ml		0.8–6.4 1.0–3.1
	Interleukin 8	Serum CSF	**32.7** pg/ml **49.6** pg/ml		6.7–16.2 15.2–38.4
	TNFα	Serum CSF	10.9 pg/ml 0.4 pg/ml		7.8–12.2 <0.5
	CXCL10	Serum CSF	76.4 pg/ml **223** pg/ml		37.2–222 6-132
	C-reactive protein		0.3 mg/l		<5
Autoimmunity	ANA		Absent		Absent
	ENA (Ro/SSA, La/SSB, SM, RNP, Scl70, Jo1)		Absent		Absent
	Anti-MPO		1 UA/ml		<11
	Anti-PR3		0 UA/ml		<11
	Serum and CSF onconeural antibodies*		Absent		Absent
	Serum neuronal surface antigen antibodies^†^		Absent		Absent
	IgLON5 antibodies		Absent		Absent
	Immunofluorescence on monkey cerebellum		Negative		Negative
SARS-CoV-2	RT-PCR on nasal swab (multiple tests)		Negative		Negative
	Serum antibodies	IgG (CLIA) IgM (CLIA) Total (ECLIA)	**43.1** UA/ml 0.5 UA/ml **275** U/ml		<8 <8 <0.79
Cerebrospinal fluid	CSF/serum glucose ratio		82%		50–90
	Total protein		334 mg/l		150–450
	Cells		0.8 /µl (100% lymphocytes)		<3
	PCR for HSV 1–2, VZV, Adeno-, Paraecho-, Enterovirus		Negative		Negative
	Borrelia and tick-borne encephalitis antibodies		Absent		Absent
	Biomarkers of neuronal degeneration or injury				
	Total Tau		**>2000** pg/ml		<404
	Phospho-Tau-181		27 pg/ml		<56.5
	β-Amyloid_1-42_		**319** pg/ml		>599
	β-Amyloid 42/40 ratio		0.088		>0.069
	Neurofilament light chain	Serum CSF	**171** pg/ml **5978** pg/ml		6.3–22.2 155–1757
	RT-QuIC for misfolded PrP		**Positive**		Negative

Abnormal results are shown in bold.

Reference ranges for interleukins in cerebrospinal fluid were obtained by our Laboratory based on 100 cerebrospinal fluid samples.

*: Onconeural antibody panel: amphiphysin, CV2, Ma2/Ta, Ri, Yo, Hu, recoverin, Sox1, titin, Zic4, GAD, Tr.

†: Neuronal surface antigen antibody panel: N-methyl-D-aspartate receptor (NMDA-R), leucine rich glioma inactivated 1 (LGI1), contactin-associated protein-like 2 (CASPR2), α-amino-3-hydroxy-5-methyl-4-isoxazolepropionic acid receptor 1 and 2 (AMPA-1-R and AMPA-2-R), γ-aminobutyric acid receptor B (GABA-B-R), dipeptidyl aminopeptidase-like protein (DPPX).

Abbreviations: ANA, antinuclear antibodies; CLIA, chemiluminescence immunoassay; CSF, cerebrospinal fluid; CXCL10, C-X-C Motif Chemokine Ligand 10; ECLIA, electrochemiluminescence immunoassay; ENA, extractable nuclear antigens; HSV, herpes simplex virus; MPO, myeloperoxidase; PCR, polymerase chain reaction; PR3, proteinase 3; PrP, prion protein; RT-PCR, reverse transcriptase polymerase chain reaction; RT-QuIC, real-time quaking-induced conversion; TNFα, tumour necrosis factor α; VZV, varicella zoster virus.

A diagnosis of probable sCJD was made based on current diagnostic criteria [3]. Full open reading frame sequencing of the *PRNP* gene showed a methionine/methionine polymorphism at codon 129 but no pathogenic mutations. The patient progressed to a bedridden state with preserved consciousness, regular sleep-wake cycles, slow and irregular voluntary gaze deviation towards the examiner and caregivers, grimacing and vocalizations on nonpainful and painful cutaneous stimulation and diffuse myoclonic movements synchronous with the electroencephalographic sharp-wave complexes. A percutaneous endoscopic gastrostomy was performed to administer enteral nutrition in the form of a ketogenic diet; he is still alive as of May 2022.

## Discussion

Our patient developed the first symptoms of sCJD two months after COVID-19. Four previous cases of sCJD after COVID-19 have been reported, highlighting a possible causal relationship [4–6]: our case shows relevant features suggesting a causal link between infection and neurodegeneration, notably the early age of onset and the two-month-long latency between COVID-19 and onset of neurological symptoms. The young age at onset and the limited familial history prompted us to perform genetic testing. We excluded pathogenic mutations and we found a homozygosity for methionine at *PRNP* codon 129, which is known to confer a higher risk of developing sCJD compared to the heterozygous state [7]. The parallel serum and CSF cytokine profile showed evidence of central nervous system inflammation (increased IL-6 and IL-8) and glial activation (increased CXCL10), without evidence of endothelial damage (normal CSF MR-ProADM level). An increase in CSF levels of IL-4, IL-8, IL-10 and/or IL-17 has been previously described in sCJD cases [8–10]; however, a pattern that includes increased IL-6 and IL-8 has been specifically observed during the COVID-19 pandemic [11]. This cytokine profile may support a post-COVID-19 neuroinflammatory status, although an inflammatory response related to prion-induced neurodegeneration cannot be excluded. Moreover, SARS-CoV-2 RBD-specific IgG were present at high titre. Triggering factors for PrPc misfolding in sCJD are still unclear, and may include stress, mutation, age and overexpression of the protein itself [12]. However, it has been shown that an influenza A strain can directly induce PrPc misfolding into PrPSc, forming infectious prions. This current line of evidence highlights a possible causative link between viral infections and induction of prion diseases [13], as already suggested for other sporadic neurodegenerative diseases such as Alzheimer disease and Parkinson disease [14,15]. We hypothesize that a COVID-19-related neuroinflammatory state acted on a predisposing genetic background to induce the misfolding and subsequent aggregation of PrP.

Overall, clinical, neurophysiological, neuroradiological and genetic features are strongly suggestive of sCJD subtype MM1 [16]. The reportedly normal brain MRI studies initially performed at another centre represent a possibly atypical feature; without the possibility of personally reviewing brain images, we cannot completely rule out the presence of early modifications that may be more easily identified in hindsight. Without a neuropathological and molecular analysis, we still cannot completely rule out a case of variant CJD (vCJD); however, clinical and neurological features and RT-QuIC positivity are highly atypical for vCJD and a possible exposure has not emerged.

Demonstrating the presence of SARS-CoV-2 RNA or proteins on post-mortem brain examination may further strengthen the link between infection and neurodegeneration. However, it has been reported that the presence of SARS-CoV-2 in the brain does not adequately distinguish patients with and without neurological manifestations after COVID-19 [17].

It has been recently demonstrated that SARS-CoV-2 spike proteins show high affinity for amyloid-forming proteins, the highest being for PrP; heparin seems to further increase the affinity in template-based models [18]. This may account for the appearance or worsening of symptoms of neurodegenerative diseases after COVID-19, although a definitive estimate of the neurological burden of COVID-19 will require the creation and analysis of dedicated prospective registries [2]. On the other hand, current world-wide CJD epidemiological data do not show an increase of CJD cases since the beginning of the COVID-19 pandemic [6,19].

In conclusion, the present case adds sCJD to the growing group of post-COVID-19 neurological diseases and emphasizes the link between neuroinflammation and protein misfolding. Further preclinical and epidemiological studies are needed to establish the actual role of SARS-CoV-2 as an initiator of neurodegeneration.

## Materials and methods

The patient underwent all diagnostic and therapeutic procedures deemed relevant based on current clinical practice. A written informed consent has been obtained from the patient’s family. A specific approval by the Regional Ethical Committee of Friuli-Venezia Giulia (Comitato Etico Unico Regionale FVG) is currently not required for single case reports. We used the CARE checklist when writing our report [20].

## Geolocation information

This study was performed at the Santa Maria della Misericordia University Hospital, Udine, Friuli-Venezia Giulia, Italy.
